# Effects of abciximab on key pattern of human coronary restenosis in vitro: impact of the SI/MPL-ratio

**DOI:** 10.1186/1471-2261-6-14

**Published:** 2006-04-04

**Authors:** Rainer Voisard, Mustafa Alan, Lutz von Müller, Regine Baur, Vinzenz Hombach

**Affiliations:** 1 Department of Internal Medicine II – Cardiology, University of Ulm, Robert-Kochstrasse 8, D-89081 Ulm, (Rainer Voisard, M.D., Mustafa Alan, Regine Baur, Vinzenz Hombach, M.D.), Germany; 2 Department of Virology, Institute of Mikrobiology and Immunology, University of Ulm, Robert-Kochstrasse 8, D-89081 Ulm, (Lutz von Müller, M.D.), Germany

## Abstract

**Background:**

The significant reduction of angiographic restenosis rates in the ISAR-SWEET study (intracoronary stenting and antithrombotic regimen: is abciximab a superior way to eliminate elevated thrombotic risk in diabetes) raises the question of whether abciximab acts on clopidogrel-independent mechanisms in suppressing neointimal hyperplasia. The current study investigates the direct effect of abciximab on ICAM-1 expression, migration and proliferation.

**Methods:**

ICAM-1: Part I of the study investigates in cytoflow studies the effect of abciximab (0.0002, 0.002, 0.02, 0.2, 2.0, and 20.0 μg/ml) on TNF-α induced expression of intercellular adhesion molecule 1 (ICAM-1). Migration: Part II of the study explored the effect of abciximab (0.0002, 0.002, 0.02, 0.2, 2.0, and 20.0 μg/ml) on migration of HCMSMC over a period of 24 h. Proliferation: Part III of the study investigated the effect of abciximab (0.0002, 0.002, 0.02, 0.2, 2.0, and 20.0 μg/ml) on proliferation of HUVEC, HCAEC, and HCMSMC after an incubation period of 5 days.

**Results:**

ICAM-1: In human venous endothelial cells (HUVEC), human coronary endothelial cells (HCAEC) and human coronary medial smooth muscle cells (HCMSMC) no inhibitory or stimulatory effect on expression of ICAM-1 was detected. Migration: After incubation of HCMSMC with abciximab in concentrations of 0.0002 – 2 μg/ml a stimulatory effect on cell migration was detected, statistical significance was achieved after incubation with 0.002 μg/ml (p < 0.05), 0.002 μg/ml (p < 0.001), and 0.2 μg/ml (p < 0.05). Proliferation: Small but statistically significant antiproliferative effects of abciximab were detected after incubation of HUVEC (0.02 and 2.0 μg/ml; p = 0.01 and p < 0.01), HCAEC (2.0 and 20.0 μg/ml; p < 0.05 and p < 0,01), and HCMSMC (2.0 and 20.0 μg/ml; p < 0.05 and p < 0.05). The significant inhibition (SI) of cell proliferation found in HCAEC and HCMSMC was achieved with drug concentrations more than 10 times beyond the maximal plasma level (MPL), resulting in a SI/MPL-ratio > 1.

**Conclusion:**

Thus, the anti-restenotic effects of systemically administered abciximab reported in the ISAR-SWEET-study were not caused by a direct inhibitory effect on ICAM-1 expression, migration or proliferation.

## Background

The observations that abciximab was associated with a reduction in angiographic restenosis rates in the ISAR-SWEET- study [1] was surprising. In previous placebo-controlled trials of abciximab during coronary intervention, GP IIb/IIIa blockade was found to reduce target vessel revascularization (TVR) rates after ballon angioplasty in patients without diabetes only in the EPIC trial [2], to have no influence on TVR in patients without diabetes after balloon angioplasty in EPILOG [3], or to reduce TVR and angiographic restenosis in patients with diabetes only after stenting in the EPISTENT [4,5] and ADMIRAL trials [6]. Moreover in the ISAR-SMART-2 trial [7] and in the CADILLAC-study [8] angiographic restenosis did not differ between patients treated with abciximab and placebo, both after angioplasty and stenting.

The significant reduction in angiographic restenosis in ISAR-SWEET [1] and CADILLAC [2] raises the question of whether abciximab acts on clopidogrel-independent mechanisms in suppressing neointimal hyperplasia. Two potential examples of such mechanisms suggested include anti-inflammatory effects on leukocyte Mac-I [9] and antiproliferative effects on vitronectin receptor on platelets and smooth muscle cells [10]. Restenosis is essentially characterized by migration and proliferation of smooth muscle cells and extracellular matrix accumulation. In human coronary restenotic lesions highly increased migratory [11] and proliferative activity [12] have been reported. There is now increasing evidence for a role of inflammation in the development of restenosis. Our group has demonstrated in a human coronary three-dimensional model of leukocyte attack (3DLA-model) that monocytes trigger a reactive proliferation of smooth muscle cells [13]. Several authors have suggested that the early rise in systemic markers of inflammation after angioplasty can be diminished by abciximab [14,15].

The current study investigates the effect of abciximab on expression of the intercellular adhesion molecule-1 (ICAM-1), migration, and proliferation in human vascular cells. The clinical relevance of the data is characterized by the so-called SI/MPL-ratio [16], calculating the relation between a significant inhibitory in vitro effect (SI) and the maximal plasma level (MPL) of abciximab in vivo. A SI/MPL-ratio < 1 characterizes an in vitro effect that can be achieved after systemic administration of an agent in vivo, a ratio > 1 indicates a mere local high dose option.

## Methods

### Cell culture

Endothelial cells from human umbilical veins (HUVEC) were isolated after vaginal delivery by enzymatic disaggregation with collagenase/dispase as described previously [17]. Endothelial cells from human coronary arteries (HCAEC) were purchased at Cambrex Bio- products (Vervier, B). Cells were cultured in Endothelium Growth Medium (Cambrex Bioproducts) and identified by the typical "cobble stone" growth pattern and positive reaction against von Willebrand factor (Dakopatts, Hamburg, D). Smooth muscle cells from the human coronary media (HCMSMC) were purchased at Cambrex Bioproducts. HCMSMC were grown in Smooth Muscle Cell Growth Medium (Cambrex Bioproducts). For identification of HCMSMC antibodies against smooth muscle α-actin (Sigma, Taufkirchen, D) were used.

### Abciximab

Abciximab: Reopro^®^, Lilly, Bad Homburg, D, 0.0002 – 20 μg/mL, dilution: aqua ad inject., MPL: 0.175 μg/mL [18].

### Flow cytometry

For flow cytometry analysis of the expression of ICAM-1 in HUVEC, HCAEC, and HCMSMC, 5 × 10^4 ^cells were seeded into 6-well dishes. Abciximab (0.0002, 0.002, 0.02, 0.2, 2.0, and 20.0 μg/ml) was added to the cultures for a period of 18 h. During the last 6 h of abciximab incubation, the expression of adhesion molecules was stimulated by adding of TNF-α (20 ng/ml).

After abciximab/TNF-α treatment, cells were washed twice with phosphate-buffered saline (pH 7.2) containing 1% fetal calf serum at 4°C. Cells were resuspended in 100 μl of a FITC-conjugated monoclonal antibody directed against ICAM-1 (clone 84H10, Dianova Immunotech; final concentration 10 μg/ml) and incubated for 20 min at 4°C. A total of 1 × 10^4 ^cells (100% gated) were analyzed immediately with a flowcytometer (BDFACsCalibur, Becton Dickinson, Heidelberg, D). Controls were carried out with IgG-FITC and actinomycin.

### Migration studies

Migration of HCMSMC was measured by a 24 well colorimetric assay (Chemicon, Hampshire, UK), based on the Boyden Chamber principle. HCMSMC were incubated with SmBM medium supplemented with 1% fetal calf serum (fcs) for a period of 48 h. Thereafter HCMSMC were seeded on the upper side of the polycarbonate membrane (pore size 8 μm) of the Boyden Chamber. Migration of HCMSMC was stimulated by filling the lower chamber of the kit with SmBM medium supplemented with 10% fcs. Abciximab was added to the medium of the lower chamber in concentrations of 0.0002, 0.002, 0.02, 0.2, 2.0, and 20.0 μg/mL. After 24 h of incubation HCMSMC were removed from the upper side of the membrane. Thereafter the membranes were stained for 20', airdryed, and incubated with extraction buffer for 15'. The opitical density of 100 μl of this solution was measured at 560 nm, SmBM medium supplemented with 10% fcs was used as control (100%).

### Proliferation studies

HUVEC, HCAEC, and HCMSMC in passages 3–5 were seeded in a density of 3 – 5 × 10^3 ^cells × cm^-2 ^in 6-well dishes. 24 h after seeding the corresponding culture medium was renewed and the number of adherent cells was analyzed in a cell counter (CASY TTC, Schärfe System, Reutlingen, D). Subsequently abciximab was added in concentrations of 0.0002, 0.002, 0.02, 0.2, 2.0, and 20.0 μg/mL for another five days. Culture medium and abciximab were renewed at day three after seeding. Cell number after incubation with abciximab was calculated as relative cell number in comparison to untreated controls with the concerning solvent. Taking into account that not all cells could be successfully cultured, cell numbers of untreated controls were calculated as:

Total cell number at day 6 – cell number attached at day 1 after seeding = 100%.

### Vitality of cells

In a luminescent cell viability assay (CellTiter-Glo^TM^, Promega, Mannheim, D) the effects of abciximab in concentrations of 0.0002, 0.002, 0.02, 0.2, 2.0, and 20.0 μg/ml (HUVEC, HCAEC, and HCMSMC) were analyzed for a period of five days in 96 well dishes (Nunc, Roskilde, DK). Luminescence of luciferase reaction as a marker of cell viability was measured in a CentroLB960 (Berthold, Technologies, Bad Wildbad, D).

### SI/MPL-Ratio

As recently reported by our group [16] a SI/MPL-ratio was calculated in order to characterize the clinical impact of positive in vitro data:

Concentration with a significant inhibition in vitro (SI)

Maximal systemic plasma level (MPL)

### Statistical analysis

Data of migration and proliferation studies are presented as mean ± S.D. Statistical significance of differences between controls and drug-treated cells was determined by paired Student's t-test. Statistical significance was accepted for *P *< 0.05.

## Results

### Identification of cells

Monocultures of HUVEC and HCAEC were identified by positive reaction with antibodies directed against von Willebrand factor and by the typical "cobblestone" growth pattern in culture. Monocultures of HCMSMC exhibited the "hill and valley" growth pattern and reacted positively with antibodies against smooth muscle α-actin.

### Effects of abciximab on expression of ICAM-1

**Figure 1:**
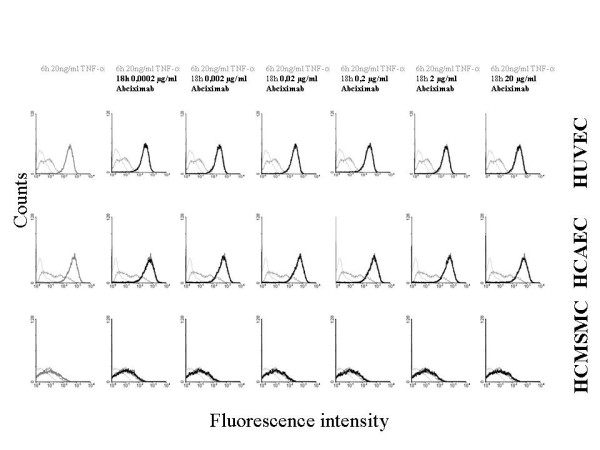
Expression of ICAM-1. The effects of abciximab (0.0002, 0.002, 0.02, 0.2, 2.0, and 20.0 μg/ml) on TNF-α induced expression of intercellular adhesion molecule 1 (ICAM-1) in HUVEC, HCAEC and HCMSMC after 18 h (cytoflow data): Controls (thin grey line), non stimulated basic expression of ICAM-1 (grey line), expression of ICAM-1 after stimulation with TNF-α (thick grey line), expression of ICAM-1 after stimulation with TNF-α plus abciximab (black line).

The effects of abciximab (0.0002, 0.002, 0.02, 0.2, 2.0, and 20.0 μg/ml) on the TNF-α induced expression of ICAM-1 are demonstrated in Figure 1. In HUVEC, HCAEC, and HCMSMC no significant effects of abciximab were detected.

### Effects of abciximab on cell migration

**Figure 2:**
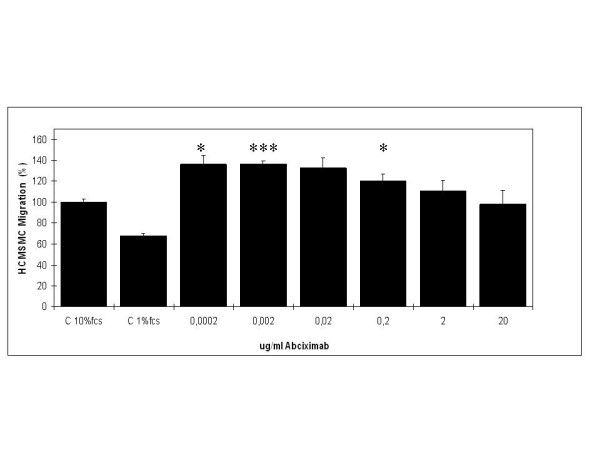
Migration. Effects of abciximab (0.0002, 0.002, 0.02, 0.2, 2.0, and 20.0 μg/ml) on migration of HCMSMC (48 h) in comparison to untreated controls. C = controls, bar 100 μm. (p < 0.05 = *; p < 0.01 = **; p < 0.001 = ***; paired Student's t-test).

The effects of abciximab (0.0002, 0.002, 0.02, 0.2, 2.0, and 20.0 μg/ml) on migration of HCMSMC are shown in Figure 2 and Table 1. After a migration period of 24 h a stimulatory effect was detected after incubation of HCMSMC with abciximab in concentration of 0.0002 μg/ml – 2 μg/ml, no effect was found after incubation with the maximal concentration of 20 μg/ml.

**Table 1:** Migration. Effects of abciximab (0.0002, 0.002, 0.02, 0.2, 2.0, and 20.0 μg/ml) on migration of HCMSMC (48 h) in comparison to untreated controls, C = controls

**Migration (%)**	HCMSMC
C	100
0,0002 μg/ml Abciximab	136,29 ± 8,66
0,002 μg/ml Abciximab	136,68 ± 2,97
0,02 μg/ml Abciximab	132,43 ± 10,01
0,2 μg/ml Abciximab	120,37 ± 6,01
2 μg/ml Abciximab	110,81 ± 10,0
20 μg/ml Abciximab	98,17 ± 13,23

After incubation of HCMSMC with abciximab in concentrations of 0.0002, 0.002, and 0.02 μg/ml cell migration was increased by 36.29% (p < 0.05), 36.68% (p < 0.001), and 32.43% (n.s.). The stimulatory effect decreased after incubation with 0.2 and 2 μg/ml of abciximab, cell migration was increased by 20.37% (p < 0.05) and 10.81% (n.s.), respectively. After incubation of HCMSMC with the maximal concentration of 20 μg/ml of abciximab no effect on cell migration was detected.

### Effects of abciximab on cell proliferation

**Figure 3:**
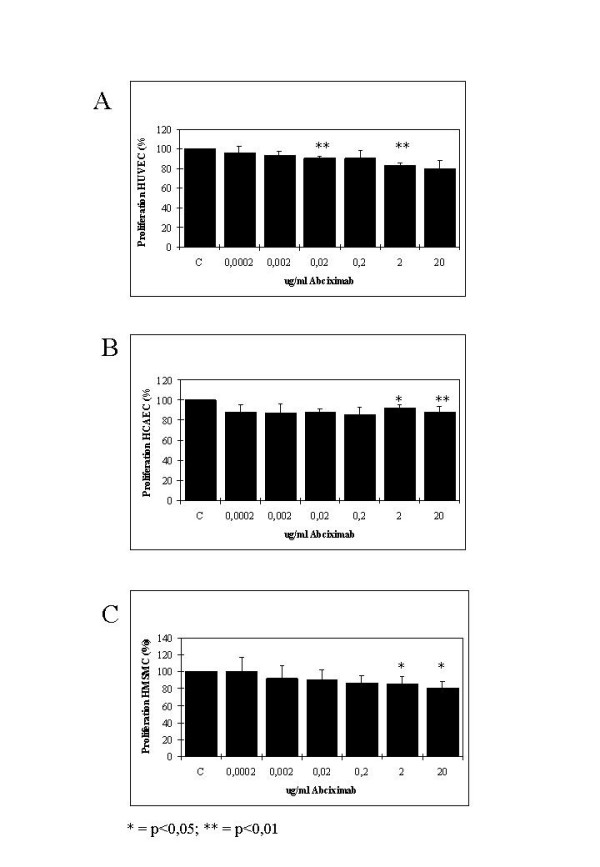
Proliferation. Effects of abciximab (0.0002, 0.002, 0.02, 0.2, 2.0, and 20.0 μg/ml) on proliferation of HUVEC (A), HCAEC (B), and HCMSMC (C) in comparison to untreated controls at day six after seeding, C = control, (p < 0.05 = *; p < 0.01 = **; p < 0.001 = ***; paired Student's t-test).

The effects of abciximab (0.0002, 0.002, 0.02, 0.2, 2.0, and 20.0 μg/ml) on proliferation of HUVEC, HCAEC, and HCMSMC are demonstrated in Figure 3 and Table 2. After incubation with high concentrations of abciximab small but significant inhibitory effects were detected.

**Table 2:** Proliferation. Effects of abciximab (0.0002, 0.002, 0.02, 0.2, 2.0, and 20.0 μg/ml) on proliferation of HUVEC, HCAEC, and HCMSMC in comparison to untreated controls at day six after seeding, C = control, (p < 0.05 = *; p < 0.01 = **; p < 0.001 = ***; paired Student's t-test)

**Proliferation (%)**	**HUVEC**	**HCAEC**	**HCMSMC**
C	100 ± 0	100 ± 0	100 ± 0
0,0002 μg/ml Abciximab	96,41 ± 6,16	88,5 ± 7,2	100,18 ± 17,10
0,002 μg/ml Abciximab	93,77 ± 3,88	87,17 ± 9,02	91,86 ± 15,64
0,02 μg/ml Abciximab	90,19 ± 1,77	88,35 ± 3,04	90,85 ± 11,33
0,2 μg/ml Abciximab	90,37 ± 7,38	85,09 ± 8,27	86,90 ± 8,43
2 μg/ml Abciximab	83,47 ± 2,51	91,87 ± 3,25	85,79 ± 8,56
20 μg/ ml Abciximab	79,34 ± 9,16	87,70 ± 6,01	81,09 ± 7,59

In HUVEC small but significant inhibitory effects were detected after incubation with abciximax in concentrations of 0.02 μg/ml and 2 μg/ml, cell proliferation was decreased to 90.19% (p = 0.01; SI/MPL-ratio: 0.11) and 83.47% (p < 0.01; SI/MPL-ratio: 11.4), respectively. No significant inhibitory effects were found after incubation of HUVEC with abciximab in concentrations of 0.0002 μg/ml, 0.002 μg/ml, 0.2 μg/ml, and 20 μg/ml, cell proliferation was decreased to 96.41%, 93.77%, 90.37%, and 79.34%.

In HCAEC no significant inhibitory effects were detected after incubation with abciximab in concentrations of 0.002 μg/ml, 0.002 μg/ml, 0.02 μg/ml, and 0.2 μg/ml, cell proliferation was decreased to 88.50%, 87.17%, 88.35%, and 85.09%. Small but significant inhibitory effects were found after incubation of HCAEC with abciximab in concentrations of 2 μg/ml and 20 μg/ml, cell proliferation was decreased to 91.87% (p < 0.05; SI/MPL-ratio: 11.4) and 87.70% (p < 0.01; SI/MPL-ratio: 114).

After incubation of HCMSMC with abciximab in concentrations of 0.002 μg/ml, 0.002 μg/ml, 0.02 μg/ml, and 0.2 μg/ml no significant inhibitory effects were detected in comparison to untreated controls, cell proliferation was 100.18%, 91.86%, 90.85%, and 86.90%. Small but significant inhibitory effects were found after incubation of HCMSMC with abciximab in concentrations of 2 μg/ml and 20 μg/ml, cell proliferation was decreased to 85.79% (p < 0.05; SI/MPL-ratio: 11.4) and 81.09% (p < 0.05; SI/MPL-ratio: 114).

### Effects of abciximab on cell vitality

Cell vitality of HUVEC, HCAEC, and HCMSMC was analyzed after incubation with abciximab in concentrations of 0.0002, 0.002, 0.02, 0.2, 2.0, and 20.0 μg/ml. Cell vitality was slightly increased in HUVEC and HCAEC and slightly decreased in HCMSMC. Statistical significance of the differences in comparison to untreated controls was not achieved.

In HUVEC cell vitality was slightly increased after incubation with abciximab in concentrations of 0.0002, 0.002, 0.02, 0.2, 2.0, and 20.0 μg/ml. Cell vitality in comparison to untreated controls was 106.83%, 117.57%, 112.30%, 108.43%, 111.31%, and 111.36%.

In HCAEC a similar result was detected, cell vitality was slightly increased. In comparison to untreated controls cell vitality was 113.38%, 117.24%, 105.99%, 112.68%, 109.15%, and 106.70%.

In HCMSMC a slight decrease of cell vitality was detected after incubation with abciximab in concentrations of 0.0002, 0.002, 0.02, 0.2, 2.0, and 20.0 μg/ml. Cell vitality in comparison to untreated controls was 86.94%, 86.51%, 96.64%, 82.50%, 97.49%, and 89.82%.

## Discussion

The present in vitro study investigated the effects of abciximab on key pattern of human coronary restenosis. Three basic conclusions were determined. First, abciximab (0.0002 μg/ml – 20 μg/ml) had no effect on expression of ICAM-1 in HUVEC, HCAEC, and HCMSMC. Second, abciximab (0.0002 μg/ml – 2 μg/ml) stimulated migration of HCMSMC. Third, high concentrations of abciximab had a small but significant antiproliferative effect in HUVEC, HCAEC, and HCMSMC, SI/MPL-ratio's > 1 indicate that these effects can't be achieved after systemic infusion.

During the last decade, intensive efforts have been made to evaluate the role of the platelet glycoprotein (GP) IIb/IIIa complex in platelet-mediated thrombus formation. Acitivation of the GP IIb/IIIa platelet-surface integrin by endogenous agonists (e.g. thrombin, adenosine diphosphate or ADP, and collagen) results in binding of adhesive proteins such as fibrinogen and von Willebrand factor, thus mediating platelet aggregation [20]. Significant efforts have been made to design potent antagonists of this "final common pathway" of platelet aggregation to be used as novel therapeutic strategies to treat acute coronary syndromes. Antagonists directed against the GP IIb/IIIa receptor represent a family of antiplatelet drugs that are used clinically for the prevention of pathological thromboses [21]. Abciximab, a monoclonal antibody Fab fragment, was the first approved agent in this class of drugs [22]. Although a decade ago abciximab has been shown to prevent acute ischemic complications from percutaneous transluminal coronary angioplasty (PTCA) and atherectomy [23], recent data of Kastrati et al. [24] in the ISAR-REACT-study did not find any clinically measurably benefits during the first 30 days in low to intermediate percutaneous coronary interventions after pretreatment with a high loading dose of clopidogrel and abciximab in comparison to clopidogrel alone.

Charo et al. [25] were the first to demonstrate that platelet GP IIb/IIIa-like proteins mediate the adherence of HUVEC to specific adhesive proteins. Yasukawa et al. [26] demonstrated that ICAM-1 is expressed early and intensely in rat carotid arteries after balloon injury and that monoclonal antibodies to ICAM-1 significantly suppress restenosis. ICAM-1 is a highly glycosylated cell surface protein of 95 kD that is expressed on variable levels on endothelial cells, smooth muscle cells, and circulating leukocytes [27]. In cultured human coronary endothelial and smooth muscle cells expression of ICAM-1 is highly upregulated by TNF-α [28]. Inoue et al. [29] demonstrated that serum levels of ICAM-1 and P-selectin were significantly increased immediately after angioplasty in human coronary sinus blood samples. Therefore inhibition of ICAM-1 has been targeted as a strategy to inhibit restenosis [30].

The current study demonstrates that abciximab in concentrations ranging from 0.0002 μg/ml – 20 μg/ml has no effect on expression of ICAM-1 in HUVEC, HCAEC, and HCMSMC. The data are in accordance with a recent report of Massberg et al. [31] that abciximab infusion did not significantly affect platelet-induced endothelial cell expression of ICAM-1 in 20 patients undergoing coronary stenting. On the other hand Schwarz et al. [32] reported that abciximab inhibited significantly adhesion of a monocytic cell line to immobilized ICAM-1 in vitro.

Migration [11] and proliferation [12] of human coronary smooth muscle cells is significantly increased in specimens derived from restenosing lesions. Systemic treatment with αvβ3 antagonists such as abciximab reduce neointima formation after injury of rat carotid [33], rabbit carotid [34], hamster carotid [35], pig coronary [36], pig carotid and femoral [37] and rabbit iliac [38,39] arteries. The mechanisms that caused the anti-restenotic effect in these studies are difficult to identify. It has been reported by Stouffer et al. [40] that vascular cell β3 integrin expression is increased after injury and that abciximab binds to cultured SMC with high affinity. Moreover, the group of Stouffer et al. [40] demonstrated that β3 activation is important for alpha-thrombin induced cell proliferation.

In the present study a stimulatory effect on cell migration was detected after incubation of HCMSMC with abciximab in concentrations of 0.0002 – 2 μg/mL, statistical significance was achieved after incubation with abciximab in concentrations of 0.0002 μg/ml, 0.002 μg/ml, and 0.2 μg/ml. Neither stimulatory nor inhibitory effects on cell migration were detected after incubation of HCMSMC with abciximab in a concentration of 20 μg/ml. In the applied migration model merely direct effects on cell migration can be studied. Indirect effects that block thrombin-induced migration cannot be considered due to the fact that thrombin is not present in the system [41]. Due to the fact that a concentration of abciximab of 0,175 μg/mL is considered as maximal plasma level (MPL) after systemic infusion [18], the present data demonstrate that direct anti-migratory effects of abciximab may not contribute to the anti-restenotic effects described in experimental [33-39] and clinical [1,2,5-7] studies. These data are in contrast to a report of Blindt et al. [42,43] describing a significant anti-migratory effect after administration of abciximab in a concentration of 33 μg/mL. Cell specific differences can be excluded because the group of Blindt et al. used human coronary smooth muscle cells as well. The contrasting results may be partially explained by the fact that Blindt et al. administered abciximab 24 h before and during migration. In the current study, as in patients, abciximab was administered merely during and not before and during the migration process.

In the present study a small but significant antiproliferative effect was detected after adding abciximab in concentrations of 2 μg/mL and 20 μg/mL, resulting in SI/MPL-ratio's beyond 1. The effect of abciximab on cell proliferation in human coronary vascular cells has already been studied by the group of Blindt et al. [42,43]. Both modest antiproliferative effects [42] and no antiproliferative effect have been reported [43]. The small but significant antiproliferative in vitro effect of abciximab in the current study was achieved with SI/MPL-ratio's of 11.4 and 114, indicating that the corresponding drug concentrations were 11.4- and 114-times, respectively, beyond the MPL of 0,175 μg/mL. Equally as described for direct anti-migratory effects, a contribution of direct antiproliferative effects of abciximab for the experimental [33-39] and clinical [1,2,5-7] anti-restenotic effects can be excluded. The small but significant antiproliferative effect of abciximab however might be used in local high dose application systems such as coated stents [44].

### Limitations of the study

The present study investigates direct effects of abciximab on ICAM-1 expression, migration and proliferation. The primary target for abciximab, however, is the platelet GPIIb/IIIa receptor, which is blocked by the antibody thereby preventing complex formation between the platelet and fibrinogen. Inhibition of platelet aggregation results in the release of multiple platelet derived growth factors capable of affecting smooth muscle proliferation and expression of adhesion molecules. Thus, the absence of platelets in the in vitro system, as well as the absence of other cellular components and immunological reactions initiated by increased platelet activity are not replicated in the present experimental protocol.

## Conclusion

Although an effect of abciximab on expression of ICAM-1 in HUVEC, HCAEC and HCMSMC was excluded in the present study, an effect of abciximab on monocytes is possible and should be confirmed in further studies. One of the possible models for the evaluation of these mechanisms might be the 3DLA-model reported earlier by our group (13).

Equally as described for direct anti-migratory effects, a contribution of direct antiproliferative effects of abciximab for the experimental and clinical anti-restenotic effects can be excluded. The small but significant antiproliferative effect of abciximab (SI/MPL-ratio > 1) indicates a local high dose option that might be of clinical use in coated stents (44).

## Competing interests

The author(s) declare that they have no competing interests.

## Authors' contributions

All authors read and approved the final manuscript. RV, RB, and VH designed the study, RV wrote the manuscript. LvM carried out the cytoflow studies, cell migration studies and cell proliferation studies were done bei MA and RB.

## Pre-publication history

The pre-publication history for this paper can be accessed here:


